# Quantitative Descriptive Analysis of Traditional Herbal and Coffee Liqueurs Made with Grape Marc Spirit (Orujo)

**DOI:** 10.3390/foods9060753

**Published:** 2020-06-05

**Authors:** Sandra Cortés-Diéguez, Carmen Otero-Cerviño, Hixinio Rodeiro-Mougán, José Antonio Feijóo-Mateo

**Affiliations:** 1 Laboratory of Agro-food Biotechnology, CITI-Tecnópole, Parque Tecnológico de Galicia, San Cibrao das Viñas, University of Vigo, 32900 Ourense, Spain; 2 CRIIGG of Spirits and Traditionally Liquours from Galicia, Pazo de Quián, 15881 Sergude Boqueixón—A Coruña, Spain; carmenoteroc@gmail.com (C.O.-C.); orujodegalicia@orujodegalicia.org (H.R.-M.); orujo@orujodegalicia.org (J.A.F.-M.)

**Keywords:** spirits, coffee, herbal, liqueurs, panel training, principal component analysis, quantitative descriptive analysis, sensory analysis, taste evaluation form

## Abstract

Orujo is a recognized traditional grape marc distillate from Galicia (NW of Spain). It is also employed as an alcohol base to elaborate coffee and herbal liqueurs and spirits. In this manuscript, quantitative descriptive analysis was applied to obtain the most important sensory terms that describe these traditional beverages. Thirteen trained panelists developed a complete sensory lexicon. Sixteen sensory descriptors (four in appearance, five in aroma, four in mouth, and three in aftertaste) were defined, valuated, and scored with the corresponding references, after elimination of hedonic, synonymous, and non-pertinent attributes according to statistical methods. The panelists evaluated a total of 464 samples in order to define their sensory profile. Panel performance was investigated showing good discriminatory ability, repeatability, and reproducibility. Principal Component Analysis (PCA) was also applied to identify the sensory descriptors that better discriminate the samples. The results obtained showed the importance of including new terms (orujo, chocolate-cocoa, floral, bitter, and astringent) in the tasting sheet, mainly in the case of coffee liqueurs to improve their sensory profile. The results of this study were useful for the development and implementation of an important tool for the corresponding regulating council in the sensory characterization and qualification of Galician liqueurs.

## 1. Introduction

One of the most important logistical and environmental problems worldwide is the generation of waste from industries, mainly from the textile, livestock, agricultural, chemical, and wood sectors [1,2,3].

In wine-producing countries, the residues of winemaking, skins, pulp, seeds, and lees, far from being a problem, represent the opportunity to obtain a product with high added value like distillates or spirits, with the most popular being the *Orujos* from Spain, the Italian *Grappas*, the French *Marcs*, and the Portuguese *Bagaçeiras* [4,5,6,7]. All these distillates have in common that they come from of the distillation, under similar technology, of the winemaking residues once fermented. With a correct storage of the raw material and a proper distillation process [8,9,10,11,12], the spirits maintain the qualities of the grape varieties from which they come from and also the singular characteristics and authenticity of a traditional product linked to a certain area [13,14,15].

The majority of these distillates are consumed, after a dilution process to reduce the ethanol content (40–45% (*v*/*v*)), with or without aging time in oak barrels [16,17,18]. However, some of them are also flavored, such as *Ouzos* and *Tsipouros* [19,20,21], or used as an alcohol base to obtain liqueurs [22]. Liqueurs are sweet and alcoholic beverages obtained mixing different raw materials (plants, herbs, spices, seeds, flowers, fruits, coffee, dairy products) with distillates of agricultural origin [23]. It is also possible to distill fruit after alcoholic fermentation and then add sugar to obtain the corresponding beverage [24].

Natural or synthetic extracts are also used, which are not always allowed and whose presence detracts from the quality of the liqueur and, above all, tradition in its production. However, there are a wide variety of studies published on the volatile compounds provided by each herb and spice, making it possible to verify the presence of extracts in liqueurs, using CG-MS through not only the content of any compound but also in the relationship that can be established between them [25,26,27,28].

The liqueur elaboration process includes different stages, maceration, infusion, and distillation, or a mix of them. In these processes, volatile compounds, responsible for the aromatic notes, and phenolic compounds, with antioxidant properties and color, are transferred to the alcohol [29,30,31,32]. Phenolic compounds have a great influence on the quality of the liqueurs, mainly in the mouth due to their bitterness and astringency notes. These compounds are also responsible for the liqueur’s appearance; however, the final color results from the addition of caramel, honey, or food-grade colorants.

Galicia (NW of Spain) is a traditional viticulture area that produces Orujo spirits from the distillation of winemaking residues. Since the grape varieties cultivated in this area are very aromatic (Albariño, Loureira, Treixadura, Godello, Mencía), the corresponding distillates have a lot of intense sensory descriptors, mainly defined by floral, vegetal, and fruity notes [33].

Besides the Orujo spirits, liqueurs made with aromatic plants, spices, and coffee are also traditionally elaborated in this area and highly valuated by their digestive and healthy properties, supporting such claims in the content of phenolic compounds and antioxidants present in the medicinal and aromatic plants used in the elaboration process [34]. According with this tradition, since 2004, coffee liqueurs and herbal liqueurs and spirits were included into the Geographical Indications of the Traditional Spirits and Liqueurs from Galicia (IGP) [35].

To protect their authenticity and establish a relationship with the geographic area and traditional practices in order to differentiate them from other similar products made in other areas, the IGP normative includes a concentration range for several chemical parameters and also a generic description of their sensory characteristics (Appendix A).

The raw materials allowed in the elaboration and generalities about the production process are fixed in the specifications of the Regulatory Council of the IGP. Mint, lemon verbena, aloysia, oregano, coriander, fennel, nutmeg, chamomile, rosemary, thyme, orange blossom, licorice, and cinnamon are considered the most traditional plants and species used to elaborate Galician liqueurs; however, production is allowed with any plant or species that is food grade. In all cases, herbal liqueurs must contain a minimum of three different plants and/or species.

In the case of coffee liqueur, it must be made with natural coffee, but no mention is made either of the species (*Arabica* or *Robusta*), the geographical origin, or the grain size (grounded or whole grain).

Taking into account the number and possible combinations of raw materials that can be used to elaborate these sweet and alcoholic beverages, the variety of existing liqueurs is very wide, showing different sensory characteristics, mainly in regard to aroma descriptors.

In the last years, several research works have been published regarding the optimal conditions of maceration, extraction of volatiles, characterization of raw materials, and volatile composition of Galician liqueurs [36]. These results allow very useful information to be obtained about the analytical composition of these traditional beverages to achieve their characterization and also identify the mainly phenolic and volatile compounds from plants, spices, coffee, and alcohol base responsible for their sensory profile.

However, despite the singular composition of the liqueurs, since the introduction of these beverages in the IGP, liqueurs and young spirits have been sensory evaluated with the same taste evaluation form that was previously defined by quantitative descriptive analysis (QDA) application to young Orujo distillates [37]. Quantitative descriptive analysis is the more complete sensory tool applied in the food industry to describe and quantify sensory attributes [38].

The introduction of traditional liqueurs in the IGP implies that QDA must be used to train again the official panel in order to define a suitable liqueur lexicon to elaborate a specific tasting sheet in order to define the new characteristics, including adequate descriptors for the characterization and qualification of these alcoholic beverages. These results will be very useful to the producers and the technicians of the regulating council in the qualified sessions of liqueurs.

In this sense, the purposes of this study were (a) to develop and validate a complete liqueur lexicon using trained sensory panelists; (b) to determine the sensory attributes that are most important in the visual, aroma, taste, and aftertaste qualities to describe these traditional liqueurs in order to elaborate a useful tasting score sheet; and finally, (c) to define the sensory profile of these traditional beverages.

## 2. Materials and Methods

### 2.1. Samples

A total of 473 liqueurs and spirits were sensory evaluated at different stages of this study. All samples were collected by the technicians of the regulating council in several distilleries and producers sited in different areas from Galicia that produce these kinds of beverages according to the rules established into the legal normative of Geographic Indication Protected of Spirits and Traditional Liqueurs from Galicia. The samples were also analyzed to evaluate the concentration of the analytical parameters in order to their certification (Appendix A). Only those samples that met all the quality control requirements (sensorial and chemical) were included in this study.

Six samples of coffee and herbal liqueurs and spirits, two of each type, were evaluated in the first part of the quantitative descriptive analysis to generate the sensory descriptors in appearance, aroma, mouth, and aftertaste.

In the second part of QDA, three samples, one of each type, randomly chosen, were evaluated to validate the lexicon generated and validated by the training panelists and to familiarize them with the scale.

Finally, and with the new taste evaluation form defined by the panelists, 464 liqueurs were sensory evaluated from 2005 to 2019. In total, 213 samples belonged to the group of coffee liqueurs, 232 were herbal liqueurs, and 19 were herbal spirits.

### 2.2. Sample Preparation

All coffee and herbal liqueurs reported an ethanol concentration of 30% alcohol by volume and herbal spirits was on average 39.5% (*v*/*v*).

Samples (20 mL), without dilution, were served individually and coded with three-digit random numbers, in tulip spirit-taster glasses at 6–8 °C. All sessions were developed in a professional tasting room composed of 20 independent tasting booths.

A plate with unsalted toasted bread and a bottle of natural water was given to each panelist to rinse the mouth between samples to clean the palate under similar conditions.

Sample presentation was randomized among all panelists during the sessions to define the sensory profile.

### 2.3. Test Design

The methodology applied in this research can be divided into three phases: Panel selection, panel training, and product testing using the QDA method. All training and sensory sessions were hosted by a panel leader and two technicians from the regulatory council as assistants.

#### 2.3.1. Panel Selection and Pre-Training

In total, 22 subjects, 13 males and 9 females, ranging in age from 31 to 55 years, all of them members of the official panel of “*Geographic Indication Protected of the Spirits and Traditional Liqueurs from Galicia*”, participated in the sessions of the selection process. They had high experience in the sensorial analysis of traditional liqueurs and a special knowledge of this kind of alcoholic beverage, because most of them were distillers and local producers. However, to take part as a panelist in a quantitative descriptive analysis, training in the specific characteristics of the product being evaluated is necessary.

The development of the different tasks included in the pre-evaluation process was similar to that carried out for beer by Elgard et al., (2019) [39].

In the first session, a pre-screening questionnaire was completed by all subjects, including demographic information, sensory knowledge, involvement in other tasting panels, and availability for sensory training mainly for the future periodic product qualification sessions.

In order to evaluate the performance of each panelist, several pre-screening tests were applied before starting the training sessions. According to Ikes et al., (2017) [40], panelists were asked to carry out a basic taste test (sweet, acid, bitter, and salty) and also to identify odorants common to liqueurs and spirits. The basic taste test, with 10 mL of water solutions of each reference in plastic cups, was performed in the first day (2 h) of pre-screening training. Odor identification was evaluated in two 1-h sessions during two consecutive days. To carry out this test, 15 samples were prepared, 11 according to previous studies [37] and 4 by individual maceration, during one week of 10 g/L of aromatic plants (chamomile, fennel and mint), 10 g/L of spices (cinnamon and pepper), 40 g/L of coffee (*Arabica*), and 40 g/L of coffee (*Robusta*) prepared in an alcoholic solution at 30% (*v*/*v*). The 15 reference samples were served in 100-mL brown glass bottles with a screw top.

In the first session, the samples were smelled by the tasters and they were asked to write the descriptor that they perceived with more intensity. Each sample only could be smelt for a maximum of 30 s. After 1 h, the panelists had an hour break and then they repeated the test one more time. This test was to measure the ability to generate sensory descriptors.

In another session, on the next day, these 15 samples were smelled again, but in this test, the panelists were given a list with the name of the products and the main attributes associated with them. They were required to choose from this list the attribute that defined each sample. With this test, it was possible to evaluate the ability of the panelists to identify sensory descriptors in the different liqueur and spirit samples.

This pre-training process aimed to obtain information about the panelists and to evaluate their initial performance. A panelist had to achieve at least 80% acuity on the taste and odorant identification.

After the results obtained during the pre-training process were evaluated, only 13 subjects, 7 males and 6 females, ranging in age from 31 to 55 years, were selected to take part in the next steps of the QDA method.

#### 2.3.2. Lexicon Development and Training with References

Lexicon development is one of the most important steps in sensory analysis [38] and an expert and trained panel must be carried out. Samples in this phase should be chosen to represent the product for sensory evaluation.

After the pre-training sessions, the 13 panelists selected were asked to freely develop, for around 1 h per sample, those terms that they considered that best described the liqueur and spirit samples. A representative collection of six samples (two herbal liqueurs, two coffee liqueurs, and two herbal spirits) were tested to identify the relevant appearance, aroma, mouth, and aftertaste attributes. These samples were evaluated over a total of three days. A great number of attributes in appearance, aroma, mouthfeel, and aftertaste were obtained. The total number of descriptors was higher in coffee liqueurs (87), mainly in aroma (29) and mouthfeel (26), due to the characteristics of this traditional beverage, different coffee origins, and other raw materials that can be added during the elaboration process (fruits, spiced, caramel, aromatic herbs). Besides, the sugar level in coffee liqueurs is usually higher than in herbal ones to balance the possible bitter and acid notes from coffee due to the presence of a high content of polyphenols [41]. The number of attributes generated for herbal spirits and herbal liqueurs were similar, 78 and 77, respectively, due to the only differences among both beverages being related to the analytical composition: Sugar level, alcohol content, and Orujo proportion (Appendix A).

Hedonic, redundant, synonyms, and inappropriate terms for descriptive analysis were then disregarded from the previous list, and the number of attributes was notably reduced [42]. In this sense, taking into account the particular characteristics of each beverage, a new list was created, including 5 terms in appearance, 20 in aroma, 11 in mouthfeel, and 14 in aftertaste. This list had too many attributes so, in order to reduce the number of sensory descriptors, the intensity (I) and frequency of citation (F) of each attribute were rated, and then, the geometric mean (GM) was calculated [43]. A summary of the sensory descriptors given by the expert panel, their geometric means, and classification is given in Table 1.

Attributes with GM > 25% were selected to include them into the official taste evaluation form to describe the traditional liqueurs. However, by round-table discussion and consensus, the panel selected and refined the attributes that best described their perceptions and the panelist agreed to include terms like fruit in aroma, spicy-caustic in mouthfeel, and coffee in aftertaste, even though they had geometrical means lower than 25%, due to their importance of these attributes to describe this kind of alcoholic and sweet beverages.

In accordance with these previous results, a new taste test evaluation form to certify liqueur samples was defined (Table 2). This taste test is divided into two columns, on the right shows the qualifying terms, the same as the original card, and on the left contains the descriptive terms, generated in the sensorial sessions by the panelists. It is necessary that a liqueur reaches 22 points in the total score of qualifying terms to obtain certification into Geographic Indication of the Spirits and Traditional Liqueurs from Galicia.

When the terms to be used were agreed upon, the judges were trained, during 10 sessions, in the descriptors finally chosen using the corresponding references in aroma, mouth, and aftertaste. Table 3; Table 4 include a complete list of the attributes, definitions, and preparation procedures for each reference. During these sessions, the judges also were familiarized with the structured 6-point line scale, where 0= no detected, 1 = weak, 2 = clear but no intense; 3 = intense note: 4 = high intense note; and 5 = very high intense note.

#### 2.3.3. Sensory Profile

Finally, 464 liqueur and spirit samples were sensorially evaluated with the QDA method using the descriptor list previously defined by the trained panelists. Sample presentation was randomized among all panelists that scored each sensory descriptor using the abovementioned 6-point-scale.

#### 2.3.4. Validation of the Panel

Panel performance was evaluated by analysis of variance (ANOVA) in order to obtain reliable results on sensory analysis and to improve the selection and training of panelists. Using the sensory descriptors included in the taste test evaluation form (Table 3), the sources of variation analyzed were the samples, panelist, and session.

#### 2.3.5. Effect of Sample (Individual Discriminatory Ability)

Thirteen sensory attributes, from the 16 evaluated, were significantly different, most of them at 99.9% of confidence (*p* < 0.0001). So, these 13 terms were the most important for describing the traditional liqueurs and for explaining their differential characteristics. The other three descriptors, aroma intensity, dense-greasy, and persistence, did not show significant differences among samples. These results suggest that differences between samples are small for these parameters, because liqueurs and spirits are very similar in global terms.

#### 2.3.6. Effect of Panelist (Panel Homogeneity)

The effect panelist was only significant for persistence in aftertaste, *p* < 0.05, reflecting the homogeneity of the panel to describe the samples with the lexicon defined and also the same interpretation of the structured scales.

#### 2.3.7. Effect of Session (Individual Scoring Reproducibility)

No significant differences between replicates in different sessions were found, which indicates that the panelist did not change the way they rated the liqueurs over time. They were capable of making reproducible judgments, i.e., the samples were evaluated in the same way in different sessions. So, the use of the attributes was consistent and reflected the reproducibility of the whole panel.

### 2.4. Statistical Analysis

All statistical analysis was performed using XLstat-Pro program for Windows (Addinsoft, New York, NY, USA). Two-way analysis of variance (ANOVA) was applied to establish whether significant difference (*p* ≤ 0.05; *p* ≤ 0.01; *p* ≤ 0.001) existed. Class of liqueurs, judges, and repetition were considered as the factors. The multiple range tests (least-squares difference (LSD)) were applied to confirm the results obtained. Pearson’s correlations between descriptive attributes and qualifying parameters were also calculated. Principal component analysis (PCA) was applied to attempt the separation of the three types of alcoholic beverage (coffee and herbal liqueurs and spirits) according to the descriptive and qualifying parameters.

## 3. Results and Discussion

This manuscript shows the first study on the quantitative descriptive analysis of traditional liqueurs and spirits made with Galician Orujo distillates, as an alcohol base, and aromatic herbs and coffee. Similar research works have been published by Donnell et al., (2007) [44] that applied sensory descriptive analysis to develop a list of descriptors for several distilled beverages and Caldeira et al., (2018) [45] that evaluated the sensory and analytical characteristics of blueberry liqueurs elaborated with wine and marc distillates.

In total, 464 samples (213 coffee liqueurs, 232 herbal liqueurs, and 19 herbal spirits) were sensory evaluated along 70 sessions from 2005 to 2019 with a maximum of 7 different samples per session. Liqueur and spirit samples were provided by different local manufacturers belonging to the *Geographic Indication Protected of Spirits and Traditional Liqueurs from Galicia* (IGP) and all of them were collected by technicians of the regulating council.

Initially, 22 panelists participated in previous selection sessions to evaluate their capacity in the sensory evaluation of distilled and liqueur beverages. Thirteen of them were selected to take part of this study. After several training sessions, during lexicon development, the panelist generated a total of 242 attributes to describe the coffee and herbal liqueurs and spirits. Finally, and after group discussion, 16 descriptors, 4 in appearance, 5 in aroma, 4 in mouth, and 3 in aftertaste, were identified by the panelists to be used in the sensory evaluation of these traditional beverages. All terms were defined, and the reference samples were selected, prepared, and scored. The new tasting sheet (Table 2) also included 10 qualifying parameters (1 in visual phase, 3 in aroma, 4 in mouth, and 2 to evaluate the general impression), which are the terms used till this moment by the official panel of the IGP, to qualify these beverages. Descriptive and qualifying parameters’ intensities were scored on a 6-point scale (ranging from 0 = no detected to 5 = very high intense). By consensus between the regulatory council technicians and panelists, a liqueur and a spirit sample must obtain a total score ≥22 in qualifying parameters to belong to the IGP and none of the individual qualifying parameters can be 0 (not detected). Taking into account the importance of qualifying terms in the sensory evaluation and their subjective definition (Table 4), it is very important evaluate the statistical correlation between qualifying and descriptive parameters.

### 3.1. Pearson’s Correlation between Descriptive and Qualifying Sensory Scores

Pearson’s correlations values (r) between the scores obtained by descriptive and qualifying parameters in sensory testing of the spirit and liqueur samples are reported in Table 5, Table 6 and Table 7.

#### 3.1.1. Coffee Liqueurs

The results in Table 5 show that all descriptor parameters, except spicy, were positively correlated with the qualifying parameters. The spicy note as a descriptor in liqueurs is mainly associated with bad quality of the distillate used as the alcohol base in the elaboration process. This perception is a consequence of the high presence of aldehydes or other volatile compounds from the first fraction of the distillate (compounds with high volatility), contributing herbaceous notes to the aroma and spicy notes in the mouth [46]. For this reason, it is justified that its presence in the liqueurs will have a negative impact in the global qualifying parameters. Visual quality showed high correlation values with all descriptive terms in appearance, mainly with transparency (0.654). This sensory descriptor was also highly correlated with the fineness, both in aroma (0.521) and in taste (0.507) and with taste persistence (0.599). This result shows the influence of subjectivity of the first view in the rest of the sensory parameters evaluated, and the importance of the visual appearance for consumer acceptance [47]. The descriptor intensity in the aroma was highly correlated with the intensity (0.695) and frankness (0.642), the corresponding qualifies’ parameters. It is possible that the absence of off-flavor (frankness) allows the perception of a more intense coffee aroma in the liqueur. The tonality and shade (brown, mahogany) were positively correlated with taste persistence (0.537) so those coffee liqueurs with these visual attributes are more positively valuated in the mouth. Similar results were obtained by González et al. (2010) [48] in a sensory analysis of multifloral honey, and also found high correlation values between the color (visual) and terms used to describe the mouthfeel phase. Spiced notes (cinnamon, vanilla) showed a higher correlation value (0.500) with aroma intensity in coffee liqueurs, more than the individual coffee descriptor. The presence of spiced notes in these liqueurs was valuated well by the panelists increasing the total score. The sensory descriptor aftertaste persistence showed high correlation values with the majority of qualifying parameters in the aroma and in the mouth.

Visual parameters, global aroma intensity, coffee aroma, and aftertaste persistence are the descriptors that have greater influence in the global valuation of the coffee liqueurs. However, among the qualifying terms, those that are related to the authenticity of the beverage, genuineness (0.867), harmony (0.852), aroma fineness (0.809), and taste quality (0.875) have greater influence on the final score.

#### 3.1.2. Herbal Liqueurs

Similar to in coffee liqueurs, only spicy notes showed a negative correlation value with all qualifying parameters (Table 6). The four sensory descriptors in the visual phase showed high correlation values with the visual quality, mainly the transparency (0.826). Brightness was positively correlated with aroma intensity (0.507), whereas tonality and shade (0.546) and aromatic herbs (0.520) were correlated with aroma fineness. Terms to describe the appearance are the first valuated by the panelist and can influence in the rest of the parameters. Herbal liqueurs with a high intense color (yellow, green, gold) are associated with more plants and spices used in the elaboration and thus a more intense aroma.

Among the sensory descriptors, only the tonality and shade (0.547) and transparency (0.505) were correlated with the total score reached by the herbal liqueur samples, showing again the importance of the visual phase in the global impression. However, all qualifying parameters showed a high correlation value (>0.8) with the total score, with visual quality (0.612) and aroma intensity (0.759) showing lower values.

#### 3.1.3. Herbal Spirits

In the case of herbal spirits (Table 7), the majority of sensory descriptors showed high correlation values with the qualifying terms, mainly with visual quality, including the higher, sweet (0.835), and dense greasy (0.747). Spicy is a sensory descriptor that in herbal spirit showed a positive correlation value, mainly with visual quality (0.637). Spicy is related to the presence of Orujo in the sample, which is higher in herbal spirits than in liqueurs. Tonality and shade showed high correlation values with all the qualifying parameters and it was one of the most important in the global valuation (0.695). Aftertaste persistence was highly correlated with all qualifying parameters, mainly with taste persistence (0.732), and it was the descriptor that showed the higher correlation with the total score (0.736).

The singular characteristics of herbal spirits (100% *Orujo* and sugar content lower than 100 g/L) imply that the term alcoholic has greater influence on the sensory quality than in the corresponding herbal liqueurs. So, this parameter showed a high positive correlation value with all qualifying descriptors, except with visual quality. All qualifying parameters showed high correlation values (>0.9) with the total score.

### 3.2. Sensory Profile

**Figure 1:**
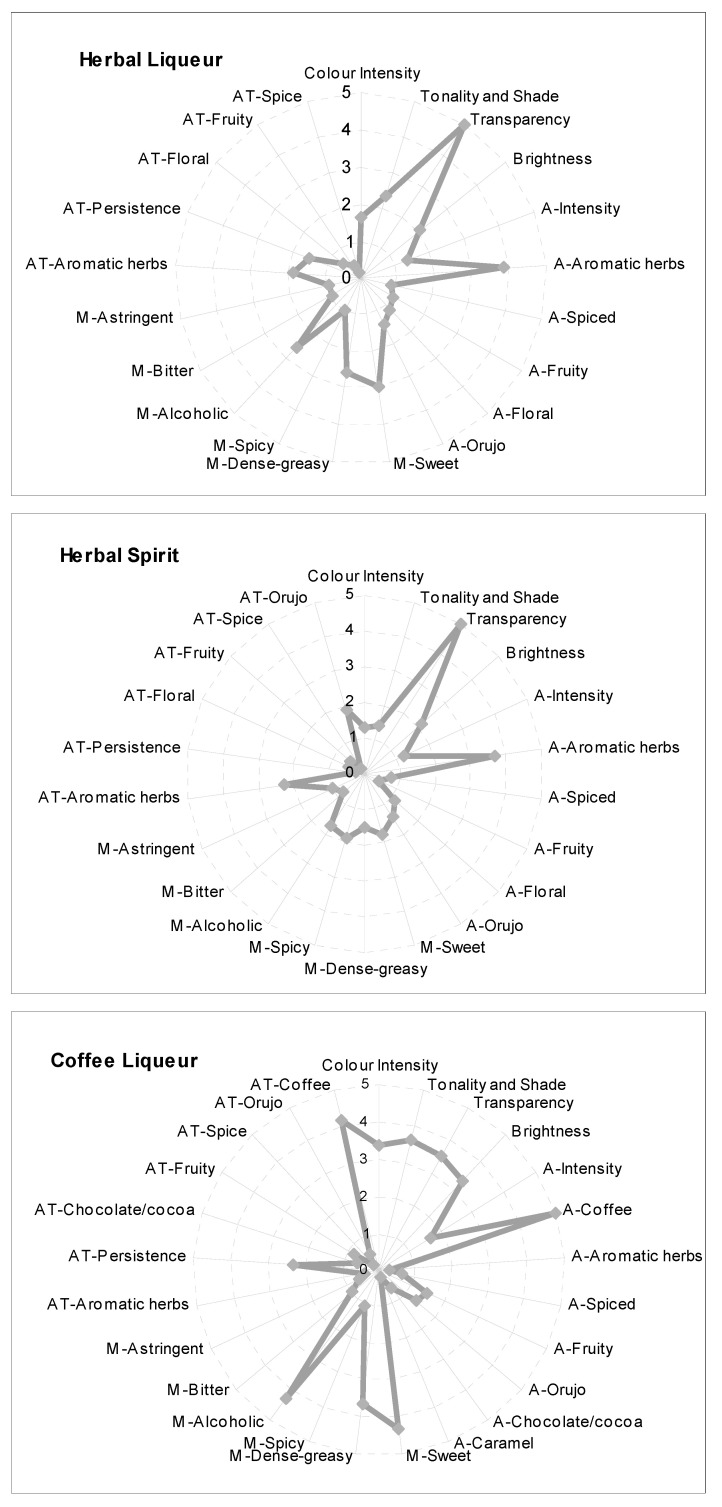
Sensory profile representing mean scores of the evaluated sensory attributes (A: aroma; AT: aftertaste; M: Mouth) for 232 herbal liqueurs; 19 herbal spirits and 213 coffee liqueurs.

Quantitative descriptive analysis was used to compare the sensory profiles of the three types of traditional coffee and herbal liqueurs and spirits. The trained panel composed by 13 trained panelists assessed 454 samples, valuing each sensory descriptor using a 6-point-scale (0= no detected, 1 = weak, 2 = clear but no intense; 3 = intense note: 4 = high intense note; 5 = very high intense note) (Table 2). The mean scores for each parameter were calculated and the results displayed in the form of spider diagrams (Figure 1).

During the sensory evaluation sessions, besides the 16 sensory descriptors included in the taste evaluation form, the panelists were free to add other attributes that they considered important to describe the sensory profile of the sample tasted. This term was scored in the column of descriptive parameters using the same 6-point scale. Taking into account the frequency and the intensity (score) of some of these terms, they were included to define the corresponding sensory profile. The new terms added for the three types of beverages evaluated were orujo in aroma, bitter and astringent in mouth and orujo, and fruity and spiced in after taste. Besides, in the case of herbal liqueurs and spirits, the descriptor “floral” was also included in aroma whereas in coffee liqueurs, terms like “caramel” and “chocolate-cocoa” were added to define the aroma and the aftertaste. Bitter and astringent are two descriptive terms also used to describe the flavor of citrus alcoholic beverages elaborated by the maceration of citrus peel in alcohol [49]. Both terms are associated with the presence of phenolic compounds in the liqueur that they were extracted from the coffee and the herbal plants during the maceration process in alcohol base (in this case Orujo) [50]. These bitter notes in some liqueurs are known, by their digestive effects, to stimulate the appetite or after a meal to aid food digestion [51].

The results in Figure 1 show that the visual parameters, mainly the tonality and shade and transparency, are very important to define herbal liqueurs and spirits, more than in coffee liqueurs due to their very high intensity brown color, which makes it difficult to evaluate the other parameters, mainly transparency. According to Castañeda-Olivares et al., (2010) [52], the visual appearance in beverages is an important determinant for consumer appearance, and is associated as an indicator of quality. The tonality and shade in herbal liqueurs and spirits depends on the plants used in the elaboration, on the ethanol concentration and titratable acidity of the alcohol base, and on the soaking time during the maceration process [53].

Herbal liqueurs and spirits are similar beverages with respect to the raw materials allowed (aromatic plants and spices) and to the elaboration process, but they are different in analytical composition (alcohol degree, sugar content, and % of “Orujo”). For this reason, their sensory profile showed a very similar graph, but with some important differences. The herbal spirit profile was more complex than the herbal liqueurs in aroma and aftertaste; however, the descriptors in the mouth had a more intense score in herbal liqueurs, probably due to less alcohol and a greater sugar content. Herbal liqueurs and spirits also included, among their descriptors, Orujo (herbaceous, grape, green), which was not included in the first lexicon developed, but obviously it has great importance in the sensory quality of these traditional beverages. Orujo was the most important to describe the herbal spirits compared to the liqueur due to Orujo being 100% of the alcohol base used in the elaboration process. Floral notes were also more intense in herbal spirits. Probably, both descriptors, orujo and floral, could be due to the alcohol base taking into account that the grape varieties in Galicia have a great number and concentration of terpenes and C13-norisoprenoids [14]. Into the descriptor of aromatic herbs, the panelists included coriander, licorice, anise, mint, chamomile, and oregano, and in the spice notes, cinnamon and nutmeg; the score intensity for this attribute was similar in both beverages. The fruity note is mainly described as being like citric and orange, because the peel of this fruit is often used in the elaboration of these beverages. In the mouth, herbal liqueurs were more balanced, alcoholic notes are equilibrated with sweet and dense and greasy descriptors, whereas herbal spirits were more alcoholic, bitter, and astringent.

In the last years, herbal and coffee liqueurs have reduced the amount of Orujo in their elaboration to make the taste of the final product smoother and to increase the aromatic notes provided by the plants and the coffee, resulting in more complex profiles.

The sensory profile of coffee liqueurs was absolutely different than those the herbal liqueurs. Coffee liqueurs displayed a high intensity of coffee notes and included, with respect to the other two beverages, descriptors like chocolate cocoa and caramel that complete their sensory profile in aroma and in aftertaste. The contribution of fruity notes was very important for the direct aroma, mainly defined as being like orange peel due to orange being traditionally used to elaborate coffee liqueurs. The mouth profile was similar to the herbal liqueurs, but with a greater intensity, with a predominance of sweet and greasy notes, resulting in a balanced beverage. Traditionally, coffee liqueurs are sweeter than herbal liqueurs in order to reduce the bitter notes from the coffee. The term alcoholic in the mouth reached a higher score than in herbal liqueurs, despite them having a similar alcohol content.

The importance that some of these terms, floral, orujo, chocolate-cocoa, bitter, and astringent, have in the sensory profile suggests the need to propose the inclusion of these new descriptors to complete the tasting test evaluation form.

### 3.3. Principal Component Analysis

To interpret the results, principal component analysis was applied. PCA helps reduce the data and allows a better graphical representation of them to be obtained, making the interpretation of multivariate analysis easier. In this study, PCA was used to identify the sensory descriptors that best discriminated among the samples analyzed.

PCA was performed with those sensory descriptors that showed significantly different intensities between the samples. In the case of coffee liqueurs, only “aroma coffee” was the descriptor with a significant mean intensity score value between samples; thus, it was not possible to apply this statistical treatment to classify the 213 samples of coffee liqueur evaluated. In this sense, the results showed that while panelists were able to describe and qualify coffee liqueurs with the new taste test evaluation form, the selected descriptors were not adequate to discriminate among samples. This result means that probably, in this area, the coffee liqueurs have similar sensory characteristics, due to their elaboration process involving fewer ingredients (mainly coffee, orujo, and sugar) than herbal liqueurs and spirits. In addition, the sensory analysis of coffee is very complex with an abundant sensory lexicon to describe it [54]. These terms will also appear in coffee liqueurs with even greater intensity due to the extraction during the maceration process. For this reason, and taking into account the high number of samples evaluated, the results showed that it will be necessary to review the lexicon in order to include new and specific terms to describe coffee liqueurs applying a new QDA process.

#### 3.3.1. Herbal liqueurs

**Figure 2:**
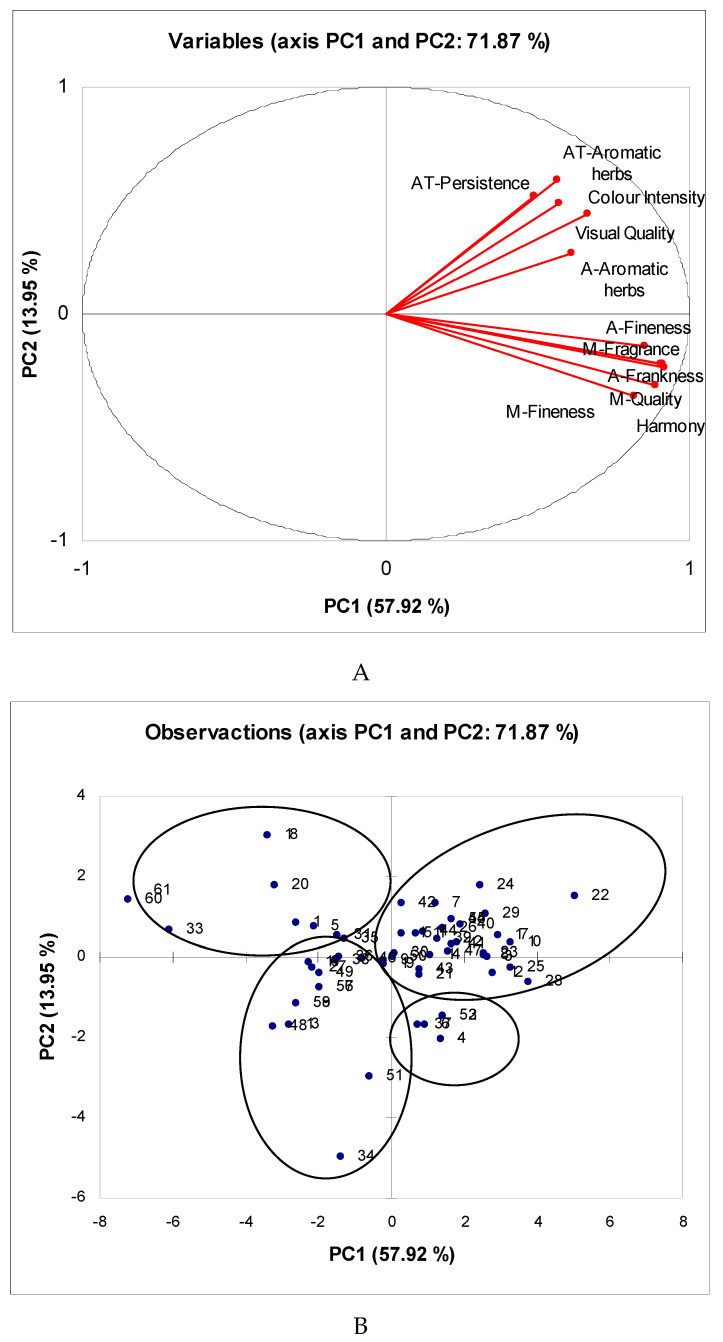
Component analysis score plot of (**A**) sensory descriptors and (**B**) samples (213 herbal liqueurs). A: aroma; AT: aftertaste; M: Mouth.

A first PCA was performed on the score of the 12 sensory descriptive and qualifying parameters with significant differences (Figure 2A). The two first principal components, PC1 and PC2, accounted for 71.87% of the total variance (57.92% and 13.95%, respectively). This percentage of variance explained was higher than the value usually obtained in the sensory analysis of food [55].

The first component (PC1) was characterized by major and positive scores of the visual quality, aroma fineness, aroma frankness, mouth-quality, mouth-fineness, mouth-fragrance, and harmony, mainly qualifying terms. For the second principal component (PC2), the persistence in aftertaste showed the highest and positive value.

In Figure 2B, four groups of samples can be observed plotted on the plane defined by the two first principal components. The majority of samples were included in group 1 and sited in the positive side of PC1 and PC2. These samples were characterized by the high scores of the sensory qualifying and descriptive parameters. In contrast, the samples in group 2, the negative side of PC1 and PC2, were herbal liqueurs with a lower score in the qualifying parameters and a lower intensity in the descriptive attributes. Samples that were disqualified in the corresponding sensory sessions belonged to group 2. Samples included in group 3, the negative side of PC2 and the positive side of PC1, were liqueur samples that were valuated well in the qualification process despite obtaining lower scores in the descriptive parameters. This group is probably formed by liqueur samples with standard characteristics, i.e., no special attributes but without a sensory defect to decide to discard them in the qualification sessions. Only a small number of samples was included in group 4, with herbal liqueurs sited in the positive side of PC2 and negative side of PC1. This group of samples obtained a good score in persistence in aftertaste, but the rest of descriptive parameters (intensity of aromatic herbs in aroma and aftertaste, color, and visual quality) and qualified parameters were valuated badly. These samples were not qualified, probably mainly due to a defect in the visual quality (turbidity or artificial color), taking into account the importance of this phase in the global qualification score.

#### 3.3.2. Herbal Spirits

**Figure 3:**
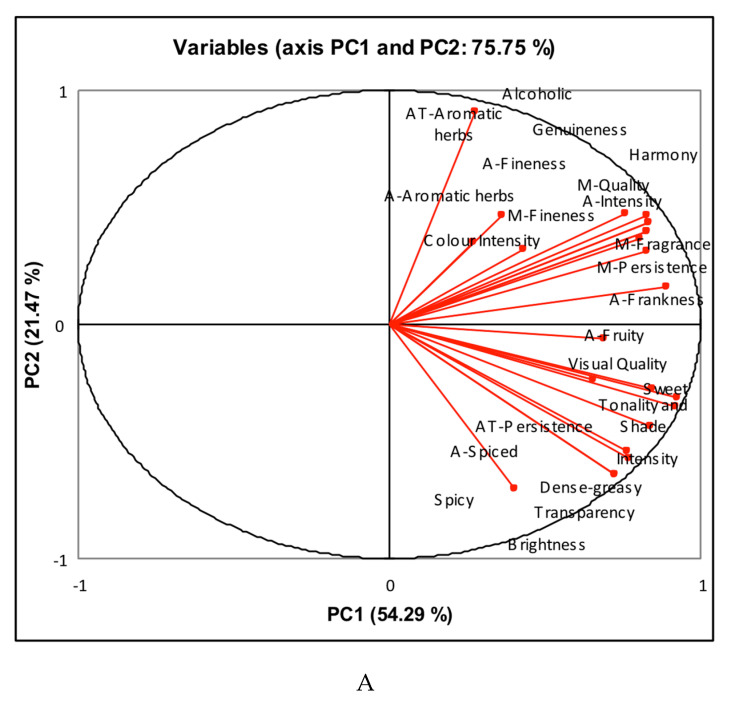

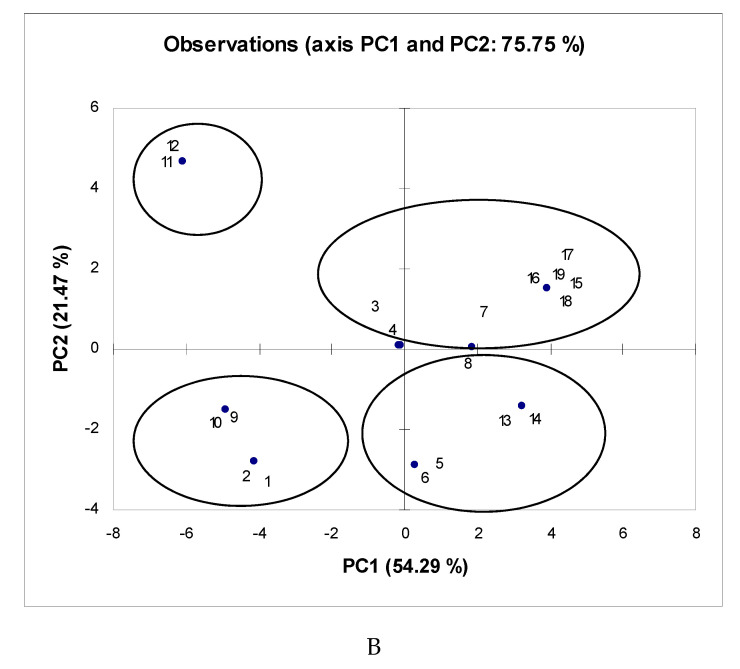
Principal component analysis score plot of (**A**) sensory descriptors and (**B**) samples (19 herbal spirits); A: aroma; AT: aftertaste; M: Mouth.

All parameters showed significant differences between the herbal spirits, so the first PCA was performed on the score of the total 24 descriptive and qualifying parameters included in the taste test evaluation form. The two first principal components, PC1 and PC2, accounted for 75.75% of the total variance (54.29% and 21.47%, respectively) (Figure 3A).

The first component (PC1) was positively correlated with the eight qualifying parameters included in the taste sheet and with the majority of the sensory descriptors, visual (tonality and shade, transparency, and brightness), aroma (intensity, spiced, and fruity), mouth (sweet, dense-greasy), and aftertaste (persistence). The second principal component (PC2) was positively correlated with alcoholic notes and negatively correlated with spicy.

The plot of Figure 3B shows a good separation of herbal spirits in four groups. The majority of samples (47%) were sited on the positive side of PC1 and PC2 and characterized by high scores of the sensory qualifying and descriptive parameters; they were the best valuated herbal liqueurs. Only two samples were included in group 2, the positive side of PC2 and negative side of PC1, characterized by alcoholic notes in the aroma. The majority of herbal spirits contain around 37.5% (*v*/*v*) of ethanol, but it is allowed reach 50% (*v*/*v*), so it is probably that those two samples in group 2 were herbal spirits with a high alcohol content that can reduce the perception of the other aromatic attributes, and for this reason, they were negatively correlated with the rest of sensory descriptors and with all qualifying parameters. Four samples were included in group 3, the negative side of PC1 and PC2, and they were positively correlated with spicy notes and negatively correlated with the rest of the attributes. These herbal spirits were probably elaborated with grape marc distillate (Orujo) with a high content of C6 compounds and aldehydes, defined by herbaceous and green notes. Four herbal spirits were belonged to group 4, the positive side of PC1 and negative side of PC2. These samples were positively correlated with the majority of descriptive parameters, mainly with those relative to the visual quality (transparency, tonality, and shade), with aroma (spiced, fruity, intensity) and mainly with those terms relative to attributes in the mouth (sweet, dense-greasy).

## 4. Conclusions

The inclusion of traditional coffee and herbal liqueurs and spirits in the Geographic Indication Protected of Spirits and Traditional Liqueurs from Galicia make necessary to have a tasting test evaluation form for their sensory description by the official tasting panel. In this sense, this study described the uses of quantitative descriptive analysis to define and validate a suitable lexicon for describing the sensory characteristics, in appearance, aroma, mouth, and aftertaste, of herbal and coffee liqueurs and spirits elaborated with Orujo (grape marc spirit from Galicia). After discarding the hedonic, synonymous, redundant, and non-pertinent attributes according to statistical methods, 13 trained panelists developed a complete lexicon, including descriptors, definitions, and references. A total of 16 sensory descriptors were approved by the panel, 4 in appearance (“color intensity”, “tonality and shade”, “transparency”, and “brightness”), 5 in aroma (“aroma intensity”, “coffee”, “aromatic herbs”, “spiced”, and “fruit”), 4 in mouth (“sweet”, “dense-greasy”, “spicy”, and “alcoholic”), and 3 in aftertaste (“aromatic herbs”, “coffee”, and “persistence”). All of them were scored using a 6-point-scale. After sensory evaluation of 464 samples (213 coffee liqueurs, 232 herbal liqueurs, and 19 herbal spirits), during 70 sessions from 2005 to 2019, the mean score for each descriptor was used to define the corresponding profile. The panel performance was also evaluated, and shown to have good discriminatory ability, repeatability, and reproducibility. The list of 16 descriptive terms was completed with other sensory descriptors like “floral” and “Orujo” and “chocolate-cocoa” and “caramel” in the aroma of herbal and coffee liqueurs, respectively, and “bitter” and “astringent” as descriptors in the mouth. These last terms were freely added, by the panelist, to the list of descriptors along the sensory sessions. PCA analysis was applied to identify the sensory descriptors that better discriminated the samples. Results showed that, in the case of coffee liqueurs, it is necessary to revise the lexicon employed to include more specific descriptive parameters for this product, taking into account that only the coffee aroma show significant differences among samples. In summary, the results obtained in this research help panelists and regulatory council technicians to describe and qualify coffee and herbal liqueurs and spirits. The results can also be extrapolated to other areas that produce similar beverages.

## Figures and Tables

**Table 1:** Classification of the sensory descriptors by their geometric means.

Descriptor	M (%) *	Class	Descriptor	M (%) *	Class
Appearance			Mouth		
**Color Intensity**	**30.4**	**4**	**Spicy - Caustic**	**24.2**	**4**
**Brightness**	**54.4**	**1**	Bitter	19.8	5
**Transparency**	**50.5**	**3**	**Sweet**	**67.9**	**1**
Clean	24.3	5	Soft	8.9	8
**Tonality and Shade**	**53.0**	**2**	Light	1.6	11
Aroma			**Dense-greasy**	**37.8**	**2**
**Intensity**	**28.5**	**2**	Astringent	10.4	7
**Aromatic plants (chamomile, coriander, rosemary, mint…)**	**62.9**	**1**	Spicy-ardent	17.7	6
**Coffee**	**26.3**	**3**	**Alcoholic**	**28.9**	**3**
**Fresh fruit (citric, stone fruit)**	**22.4**	**4**	Drying	2.6	10
Toasted-high-roast	2.9	15	Caustic	4.7	9
Floral	11.8	8	Aftertaste		
Vegetable/herbaceous	18.3	6	**Aromatic plants**	**32.5**	**2**
Sulphur compounds	3.6	14	**Persistence**	**37.6**	**1**
Honey	5.6	11	**Coffee**	**24.1**	**3**
Alcoholic	11.9	7	Fresh fruit	12.2	4
Metal	2.4	16	Floral	5.1	8
Tobacco	1.5	17	Honey	5.8	7
Orujo (Grape marc distillate)	11.0	9	Toasted-high-roast	4.8	9
**Spice (vanilla, cinnamon,**	**23.6**	**5**	Orujo	9.4	5
Balsamic	7.0	10	Impurity (mold, dirt)	4.8	9
Dried fruit (nut, hazelnut)	1.5	17	Sulphur compounds	2.4	12
Cocoa/chocolate	5.1	12	Burnt	1.4	13
Persistence	4.3	13	Vegetal/herbaceous	5.9	6
Hydrocarbon	1.1	18	Cocoa/chocolate	4.3	10
Paper	1.1	18	Spice	2.9	11

* M (%) mean of geometric means.

**Table 2:** The final taste test evaluation form used in the sensory analysis of the herbal spirits, herbal liquors, and coffee liqueurs.

Code Judge:							Sample:						
**Descriptive Parameters**							**Qualifying Parameters**						
**Appearance**	**0**	**1**	**2**	**3**	**4**	**5**	**Visual Phase**	**0**	**1**	**2**	**3**	**4**	**5**
Color Intensity							Quality						
Tonality and Shade													
Transparency													
Brightness													
**Aroma**	**0**	**1**	**2**	**3**	**4**	**5**	**Aroma**	**0**	**1**	**2**	**3**	**4**	**5**
Intensity							Intensity						
Coffee							Fineness						
Aromatic herbs							Frankness						
Spiced													
Fruity													
**Mouth**	**0**	**1**	**2**	**3**	**4**	**5**	**Mouth**	**0**	**1**	**2**	**3**	**4**	**5**
Sweet							Quality						
Dense greasy							Persistence						
Spicy							Fineness						
Alcoholic							Fragrance						
**Aftertaste**	**0**	**1**	**2**	**3**	**4**	**5**	**General Impression**	**0**	**1**	**2**	**3**	**4**	**5**
Aromatic herbs							Harmony						
Coffee							Genuineness						
Persistence							**Total score:**						

**Table 3:** List of final attributes definitions, references, and references’ preparation to train the panel.

Descriptors	Definition	Reference	Preparation
Appearance			
Color Intensity	Is the appearance that a substance has as a result of the way in which it reflects light	-	-
Tonality and Shade	A type or degree of a color	-	-
Transparency	Quality that a substance has when you can see through it	-	-
Brightness	A substance that is shining strongly or is full of light	-	-
Aroma			
Intensity	Strength of the stimuli perceived by the nose or by olfactory receptors via retronasal way	-	-
Coffee	Aroma associated with coffee	Natural coffee beans (*Coffea arabica* and *Coffea robusta*) from different origins: Brazil, Colombia, Ethiopia, Uganda and Vietnam	For each type and origin, 4 g of coffee beans in 100 mL of ethanol/H_2_O 30% (*v*/*v*) solution. Serve in 100-mL brown glass bottles with a screw top.
Aromatic plants	Aroma associated with aromatic plants	Dried natural plants, such as: mint, aloysia, oregano, coriander, fennel, chamomile, rosemary, thyme, orange blossom and licorice	For each plant, 1 g of leaves, seeds or flowers in 100 mL of ethanol/H_2_O 30% (*v*/*v*) solution. Serve in 100-mL brown glass bottles with a screw top.
Spiced	Aroma associated with spices	Ground nutmeg, dried bark strip of cinnamon, beans of vanilla and dried flowers of clove	Solutions of 1 g from each spice in 100 mL of ethanol/H_2_O 30% (*v*/*v*) solution. Serve in 100 mL brown glass bottles with screw- top.
Fruity	Aroma associated with fruit	Citrus (lemon, orange…)	A drop of citral (1 g/100 g ethanol) on a cotton ball into a glass bottle with a screw top.
		Ripe (peach, apricot…)	A drop of γ-Decalactone (1 g/100 g ethanol) on a cotton ball into a glass bottle with screw-top.
***Mouth***			
Sweet	Sensations produced by aqueous solutions of several products such as sucrose or fructose	Sucrose	Solutions of sucrose (5–10 g/L) in water. Serve in 30-mL cups cover
Dense-Unctuous	Sensation produce in the oral cavity related to resistance to fluid	Condensed milk	1 teaspoon of condensed milk. Serve in 30-mL cups.
Spicy	Trigeminal sensation in mouth that produce heat and piquancy similar to eat pepper	Ground pepper	2 g of ground pepper in 100 mL of ethanol/H_2_O 30% (*v*/*v*) solution. Serve in 100 mL brown glass bottles with screw- top.
Alcoholic	Burning sensation in the mouth associated with 40% or greater alcohol	Grape marc distillate	100 mL of grape marc distillate at 45%(*v*/*v*)
***Aftertaste***			
Coffee	Aftertaste associated with coffee	Dark roast coffee beans (*Coffea arabica*)	2 teaspoons of coffee, in teabag, in 250 mL of boiling water for 5 min. Serve in 30-mL cups cover.
Aromatic Plants	Aftertaste associated with aromatic plants	A blend of aromatic plants, such as mint, oregano, fennel, thyme….	3 g of a blend of plants, in teabag, in 250 mL of boiling water for 5 min. Serve in 30-mL cups cover
Persistence	Flavor sensation similar to that which was perceived whilst the product was in the mouth and while continues for a measurable period of time	-	-

**Table 4:** Attributes selected to qualify the global quality in liqueurs and their corresponding definitions.

Descriptors	Definition
Appearance	
Visual Quality	Describe a product without particles and/or precipitates. Clean and with the expected color.
Aroma	
Intensity	Describes the strength of the aroma
Fineness	The quality of having delicate notes
Frankness	Honest, sincere, a product without defects or strange aromas
Mouth	
Quality	Always in positive. Means how good is a product, to show a high standard
Persistence	The quality of being persistent or longer in the time
Fineness	The quality of being delicate
Fragrance	A sweet or pleasant smell
Global impression	
Harmony	A pleasant sensation made by a good combination of different sensory perceptions
Genuineness	The quality of being real and exactly what it appears to be. A liqueur that show the sensory characteristics that define these traditional beverages

**Table 5:** Pearson correlation values (R) between descriptive and qualifying parameters in coffee liqueurs.

Variables	Visual Quality	Aroma Intensity	Aroma-Fineness	Aroma-Frankness	Mouth-Quality	Mouth-Persistence	Mouth -Fineness	Mouth -Fragrance	Harmony	Genuineness	Score
Color Intensity	**0.543**	0.264	0.262	0.237	0.307	0.375	0.230	0.244	0.290	0.352	0.388
Tonality and Shade	**0.564**	0.494	0.463	0.491	0.340	**0.537**	0.367	0.363	0.384	0.367	**0.544**
Transparency	**0.654**	**0.509**	**0.521**	0.491	0.449	**0.599**	**0.507**	0.452	**0.511**	0.469	**0.643**
Brightness	**0.557**	0.476	0.361	0.453	0.331	**0.535**	0.363	0.357	0.396	0.347	**0.525**
Intensity	0.393	**0.695**	**0.523**	**0.642**	**0.502**	**0.574**	0.418	0.435	0.486	0.475	**0.645**
Aroma-Coffee	0.257	0.427	0.361	0.463	0.411	0.382	0.365	0.376	0.458	0.485	**0.501**
Aroma-Aromatic herbs	0.137	0.321	0.203	0.362	0.194	0.328	0.347	0.194	0.313	0.145	0.319
Aroma-Spiced	0.236	**0.500**	0.312	0.426	0.377	0.450	0.456	0.295	0.424	0.295	0.473
Aroma-Fruity	0.198	0.387	0.277	0.319	0.239	0.311	0.434	0.203	0.358	0.142	0.353
Sweet	0.275	0.328	0.307	0.391	0.257	0.400	0.290	0.314	0.272	0.235	0.384
Dense-greasy	0.266	0.183	0.224	0.232	0.254	0.187	0.297	0.121	0.326	0.276	0.295
Spicy	−0.060	−0.042	0.047	0.083	−0.065	0.101	0.096	−0.092	−0.085	−0.100	−0.017
Alcoholic	0.425	0.322	0.276	0.297	0.245	0.404	0.333	0.197	0.288	0.284	0.381
Aftertaste-Coffee	0.071	0.090	0.160	0.132	0.170	0.212	0.157	0.167	0.215	0.239	0.204
Aftertaste-Aromatic herbs	0.206	0.332	0.322	0.299	0.331	0.468	0.372	0.230	0.394	0.295	0.405
Aftertaste-Persistence	0.361	0.439	**0.570**	**0.541**	**0.602**	**0.653**	**0.520**	0.470	**0.560**	**0.522**	**0.655**
**Score**	**0.679**	**0.763**	**0.809**	**0.787**	**0.875**	**0.802**	**0.809**	**0.765**	**0.852**	**0.867**	**1.000**

Pearson’s correlation coefficients *r* ≥ 0.5 are given in bold.

**Table 6:** Pearson correlation values (R) between descriptive and qualifying parameters in herbal liqueurs.

Variables	Visual Quality	Aroma Intensity	Aroma Fineness	Aroma Frankness	Mouth Quality	Mouth Persistence	Mouth Fineness	Mouth Fragrance	Harmony	Genuineness	Score
Color Intensity	**0.630**	0.311	0.457	0.267	0.250	0.213	0.236	0.291	0.311	0.284	0.380
Tonality and Shade	**0.676**	0.495	**0.546**	0.466	0.406	0.437	0.354	0.414	0.431	0.440	**0.547**
Transparency	**0.826**	0.477	0.416	0.396	0.323	0.345	0.322	0.417	0.448	0.354	**0.505**
Brightness	**0.641**	**0.507**	0.400	0.365	0.369	0.351	0.393	0.447	0.426	0.386	0.499
Intensity	0.454	0.453	0.258	0.307	0.199	0.309	0.053	0.256	0.209	0.150	0.308
Aroma-Aromatic herbs	0.417	0.329	**0.520**	0.408	0.434	0.209	0.432	0.502	0.413	0.338	0.471
Aroma-Spiced	0.360	0.198	0.375	0.353	0.392	0.259	0.346	0.410	0.296	0.303	0.385
Aroma-Fruity	0.305	0.330	0.272	0.307	0.320	0.302	0.247	0.350	0.304	0.256	0.348
Sweet	0.440	0.133	0.356	0.243	0.202	0.189	0.218	0.265	0.307	0.238	0.305
Dense-greasy	0.373	0.162	0.402	0.374	0.378	0.315	0.361	0.413	0.448	0.382	0.424
Spicy	0.024	−0.061	0.136	−0.006	−0.069	−0.002	0.043	−0.110	−0.140	−0.091	−0.031
Alcoholic	0.240	0.292	0.272	0.259	0.276	0.371	0.145	0.261	0.208	0.283	0.304
Aftertaste-Aromatic herbs	0.443	0.251	0.302	0.397	0.360	0.157	0.289	0.389	0.388	0.336	0.390
Aftertaste-Persistence	0.452	0.331	0.290	0.483	0.411	0.330	0.213	0.478	0.441	0.387	0.448
**Score**	**0.612**	**0.759**	**0.847**	**0.934**	**0.921**	**0.824**	**0.850**	**0.914**	**0.942**	**0.932**	**1.000**

Pearson’s correlation coefficients *r* ≥ 0.5 are given in bold.

**Table 7:** Pearson correlation values (R) between descriptive and qualifying parameters in herbal spirits.

Variables	Visual Quality	Aroma Intensity	Aroma Fineness	Aroma Frankness	Mouth Quality	Mouth Persistence	Mouth Fineness	Mouth Fragrance	Harmony	Genuineness	Score
Color Intensity	**0.723**	**0.645**	**0.543**	0.387	0.258	0.224	0.241	0.241	0.241	0.258	0.422
Tonality and Shade	**0.741**	**0.602**	0.482	**0.741**	**0.582**	**0.642**	**0.576**	**0.609**	**0.609**	**0.582**	**0.695**
Transparency	**0.526**	0.298	0.227	**0.512**	0.359	0.444	0.367	0.398	0.398	0.359	0.437
Brightness	**0.526**	0.298	0.227	**0.512**	0.359	0.444	0.367	0.398	0.398	0.359	0.437
Intensity	**0.679**	0.484	0.306	**0.655**	0.436	**0.567**	0.407	**0.543**	**0.543**	0.436	**0.568**
Aroma-Aromatic herbs	**0.596**	**0.531**	**0.529**	0.174	0.174	0.000	0.054	0.162	0.162	0.174	0.284
Aroma-Spiced	0.465	0.342	0.319	**0.650**	0.455	**0.563**	**0.505**	0.465	0.465	0.455	**0.531**
Aroma-Fruity	0.339	**0.545**	0.221	0.436	**0.509**	**0.630**	0.385	**0.701**	**0.701**	**0.509**	**0.559**
Sweet	**0.835**	**0.562**	0.448	**0.658**	0.493	0.475	0.392	0.563	0.563	0.493	**0.614**
Dense-greasy	**0.747**	0.424	0.459	**0.546**	0.436	0.378	0.475	0.339	0.339	0.436	**0.515**
Spicy	**0.637**	0.033	0.197	0.361	0.000	0.000	0.112	−0.112	−0.112	0.000	0.124
Alcoholic	−0.068	**0.666**	**0.561**	0.327	**0.655**	**0.567**	**0.611**	**0.611**	**0.611**	**0.655**	**0.594**
Aftertaste Aromatic herbs	**0.548**	**0.604**	**0.654**	0.415	0.311	0.180	0.226	0.290	0.290	0.311	0.433
Aftertaste Persistence	**0.628**	**0.612**	**0.538**	**0.846**	**0.611**	**0.732**	**0.658**	**0.628**	**0.628**	**0.611**	**0.736**
Score	0.446	**0.912**	**0.891**	**0.908**	**0.968**	**0.909**	**0.935**	**0.923**	**0.923**	**0.968**	**1.000**

Pearson’s correlation coefficients *r* ≥ 0.5 are given in bold.

## Appendix A

**Table A1:** Quality control requirements (chemical and sensorial) established for the three types of beverages, according to the Regulating Council rules.

	Herbal Spirit		Herbal Liqueur		Coffee Liqueur	
	Maximun	Minimum	Maximun	Minimum	Maximun	Minimum
Alcohol degree (% *v*/*v*)	50	37.5	40	20	40	20
Methanol (g HL^−1^ a.a.) ^1^	950	200	950	50	950	50
Total acidity (in acetic acid, g HL^−1^ a.a.)	150	-	150	-	-	-
Acetaldehyde (g HL^−1^ a.a.)	150	-	150	-	150	-
Ethyl acetate (g HL ^−1^ a.a.)	250	-	250	-	250	-
Higher alcohols ^2^ (g HL^−1^ a.a.)	600	225	600	60	600	60
Cu (mg L^−1^)	9	-	9	-	9	-
Total sugars (gL^−1^)	100	-	-	100	-	100
Appearance	Translucent, clean, with colour from yellow pale to green		Translucent, clean, with colour from yellow pale to green		Clean, with colour from roasted amber to roasted mahogany	
Aroma	Intense, fine, delicate with floral notes, high intensity of *Orujo*, absence of musty smell, burning, dirt and acetic notes		Intense, fine, delicate with floral notes, low intensity of *Orujo*, balsamic, aromatic herbs, absence of moisture, burning, dirt and acetic notes		Intense, fine, delicate with hints of freshly roasted coffee, cocoa and chocolate, bottom of a distilled spirits of fine and elegant *Orujo*	
Mouthfeel	Persistent, balanced, no alcoholic		Sweet		Sweet, dense, enveloping, creamy, persistent. It can also present a bitter but elegant nuance	
Aftertaste	Floral, aromatic herbs and spices notes		Aromatic herbs and balsamic notes		Coffee and caramel notes, and subtle notes of chocolate or cocoa	

^1^ a.a. = absolute alcohol. ^2^ 2-butanol, 1-butanol, 1-propanol, 2-methyl propanol, 2-methyl-1-butanol and 3-methyl-butanol.
